# Interaction effect of job insecurity and role ambiguity on psychological distress in Japanese employees: a cross-sectional study

**DOI:** 10.1007/s00420-018-1288-5

**Published:** 2018-01-11

**Authors:** Akiomi Inoue, Norito Kawakami, Hisashi Eguchi, Akizumi Tsutsumi

**Affiliations:** 1 Department of Public Health, Kitasato University School of Medicine, 1-15-1 Kitasato, Minami-ku, Sagamihara, 252-0374 Japan; 2 Department of Mental Health, Graduate School of Medicine, The University of Tokyo, 7-3-1 Hongo, Bunkyo-ku, Tokyo, 113-0033 Japan

**Keywords:** Japan, Job insecurity, K6 scale, Role stress, Uncertainty

## Abstract

**Purpose:**

We examined the interaction effect of job insecurity (JI) and role ambiguity (RA) on psychological distress in Japanese employees.

**Methods:**

Overall, 2184 male and 805 female employees from two factories of a manufacturing company in Japan completed a self-administered questionnaire comprising the scales measuring JI (Job Content Questionnaire), RA (National Institute for Occupational Safety and Health Generic Job Stress Questionnaire), psychological distress (K6 scale), and potential confounders (i.e., age, education, family size, occupational class, and work shift). Taking psychological distress as a dependent variable, hierarchical multiple regression analyses were conducted by gender and employment status (i.e., permanent and non-permanent employees). An interaction term of JI × RA was included in the model.

**Results:**

After adjusting for potential confounders, the main effects of JI and RA on psychological distress were significant regardless of gender or employment status. Furthermore, the significant interaction effect of JI × RA on psychological distress was observed among permanent male employees (*β* = 0.053, *p* = 0.010). Post hoc simple slope analyses showed that the simple slope of JI was greater at higher levels of RA (i.e., one standard deviation [SD] above the mean) (*β* = 0.300, *p* < 0.001) compared to lower levels of RA (i.e., one SD below the mean) (*β* = 0.212, *p* < 0.001). On the other hand, the interaction effect of JI × RA was not significant among permanent or non-permanent female employees.

**Conclusions:**

The present study suggests that higher levels of RA strengthen the association of JI with psychological distress, at least among Japanese permanent male employees.

## Introduction

In Japan, due to a prolonged economic recession since the early 1990s, the perception of job security has declined among many employees (Tsutsumi 2016). ‘Job insecurity (JI)’ is a subjective perception of a potential threat to the continuity of the current job. Greenhalgh and Rosenblatt (1984), who performed the first study on JI, defined it as “the perceived powerlessness to maintain the desired continuity in a threatened job situation”. Since then, many other definitions have been put forward. Among others, Hellgren et al. (1999) have divided JI into two main dimensions, quantitative JI and qualitative JI. Quantitative JI is defined as “the perceived threat of job loss and the worries related to that threat” (De Witte 2005). Qualitative JI is defined as “the perceived threat of impaired quality in the employment relationship, such as deterioration of working conditions, lack of career opportunities, and decreasing salary development” (Hellgren et al. 1999). Because most studies on the association of JI with employees’ well-being have focused on the quantitative JI (De Witte et al. 2010), the present study will also concentrate on this dimension. It should be noted that permanent (or regular) employees, who are not likely to be easily laid off, are also not exempt from JI because a company may choose to lay them off if it can no longer afford to protect them due to intense competitive pressure (Kuroki 2012).

Some theoretical perspectives can explain the negative psychological consequences of JI. For example, Jahoda’s (1982) ‘latent deprivation model’ has suggested that the possibility of losing one’s job threatens the satisfaction of needs, such as income and social contacts, and leads to frustration. Furthermore, Warr’s (1984) ‘vitamin model’ has suggested that JI has a negative effect on employees’ well-being due to the associated feelings of unpredictability and uncontrollability. This theoretical relationship between JI and psychological well-being has been epidemiologically demonstrated in the occupational health research field in which several meta-analytic studies have reported the association of JI with poor mental health, such as common mental disorders and depressive symptoms (Cheng and Chan 2008; Stansfeld and Candy 2006; Sverke et al. 2002; Theorell et al. 2015). A more recent systematic review has also reported that JI is strongly associated with depressive symptoms (Kim and von dem Knesebeck 2016).

On the other hand, role ambiguity (RA) has also attracted attention as one of the classical psychosocial determinants of employee health (Hurrell and McLaney 1988). RA has been defined as “the extent to which clarity regarding job performance expectations, methods for carrying out the job, and consequences of performance is lacking” (Rizzo et al. 1970). Based on this definition, RA (or lack of role clarity [RC]) is considered, at least theoretically, one of the major stressors at work, because it imposes high cognitive overload on employees who must continuously expend energy to seek appropriate ways to accomplish their job (Fisher and Gitelson 1983; Jackson and Schuler 1985). This, in turn, reduces their psychological well-being as well as the ability to perform effectively. In fact, previous meta-analytic studies have reported the association of RA with depression (Schmidt et al. 2014) as well as with lower job satisfaction (Abramis 1994; Shen 2005), job performance (Abramis 1994), and organizational citizenship behavior (Eatough et al. 2011). A more recent systematic review has also reported that RA is associated with a greater risk of developing common mental health problems (Harvey et al. 2017).

As described above, separate studies have examined the association of JI and RA with poor mental health. On the other hand, Fried et al. (2003) pointed out that JI and RA tap the underlying construct of ‘uncertainty’ at two different levels in which JI focuses on organization-related uncertainty, whereas RA focuses on job-related uncertainty. Therefore, if employees are in uncertain situation in terms of both their own organization and job, they may be more psychologically distressed. More specifically, the association of JI with psychological distress may be greater when employees perceive higher levels of RA, while it may be weaker when they perceive lower levels of RA (or higher levels of RC). In fact, Hobfoll’s (1989) ‘Conservation of Resources (COR)’ theory suggests that RC has been identified as a supportive working condition that facilitates comprehension of employees’ work responsibilities (Panaccio and Vandenberghe 2011); therefore, it is possible that clear expectations and instructions reduce uncertainty in the workplace and consequently incite feelings of control over an insecure situation. To the best of our knowledge, however, the interaction effect of JI × RA on mental health has not been fully examined.

The purpose of the present study was to examine the interaction effect of JI × RA on psychological distress in Japanese employees. It was hypothesized that the association of JI with psychological distress would be greater among those who perceived higher levels of RA than among those who perceived lower levels of RA. Especially in the Japanese society, a traditional gender-role ideology that men are expected to be the primary breadwinners still persists (Katsurada and Sugihara 2002), which is specifically linked to gender differences in experiences and perceptions of JI, with men feeling greater insecurity compared to women (Charles and James 2005). Furthermore, the insecure situation is quite different between permanent and non-permanent employees (Virtanen et al. 2002). Therefore, we conducted statistical analyses by gender and employment status.

## Methods

### Study design

In the present study, we used a part of cross-sectional data collected from the baseline survey of an occupational cohort study on social class and health in Japan (Japanese Study of Health, Occupation, and Psychosocial Factors Related Equity: J-HOPE). More detailed information on the J-HOPE baseline survey is provided elsewhere (Inoue et al. 2014). The analyses were conducted using the J-HOPE first wave dataset as of December 22, 2016. Research Ethics Committee of the Graduate School of Medicine and Faculty of Medicine, The University of Tokyo (No. 2772-(4)), Kitasato University Medical Ethics Organization (No. B12-103), and Ethics Committee of Medical Research, University of Occupational and Environmental Health, Japan (No. 10-004) reviewed and approved the aims and procedures of the present study.

### Participant recruitment

All the employees from two factories of a manufacturing company in Japan (*n* = 3630) were recruited by means of an invitation letter sent by the authors in February 2011. All the variables used in the present study, except employment status, which was obtained from the personnel records of the surveyed company, were measured using a self-administered questionnaire. The survey was conducted from March to June 2011.

### Measures

#### Exposures: job insecurity (JI) and role ambiguity (RA)

JI was measured using a subscale of the Japanese version of the Job Content Questionnaire (JCQ) recommended version (Haratani 1997; Karasek 1985). The JCQ includes a four-item general JI scale (see Appendix). In this sample, Cronbach’s alpha coefficient was 0.50, indicating low reliability. In fact, the JCQ Center, which authorizes the use of the JCQ, has acknowledged the low reliability of the JI scale and explained this phenomenon with the fact that it collects two types of information on (i) JI and future career prospects and (ii) layoff and work instability history (see http://www.jcqcenter.org/FAQs.html). Although we tried to drop some items to achieve more statistically homogenous scale, we could not obtain a scale with better reliability (i.e., Cronbach’s alpha coefficient > 0.70). Furthermore, the JCQ Center has suggested that this approach would decrease the robustness of the scale’s interpretability. Therefore, we calculated the total score using the original four items rather than dropping some items. According to the JCQ user’s guide (Karasek 1985), the total score ranges from 4 to 17, with a higher score indicating a more insecure situation.

RA was measured using the Japanese version of the National Institute for Occupational Safety and Health Generic Job Stress Questionnaire (NIOSH-GJSQ) (Haratani et al. 1996; Hurrell and McLaney 1988). The NIOSH-GJSQ includes a six-item RA scale. Example items are, “I feel certain about how much authority I have” and “There are clear, planned goals and objectives for my job”. Items were assessed on a seven-point scale ranging from 1 = Very inaccurate to 7 = Very accurate (Rizzo et al. 1970). The total score, ranging from 6 to 42, was calculated by summing the reversed scores for each item, with a higher score indicating a more ambiguous situation. The English version of the NIOSH-GJSQ was translated into Japanese language, and the internal consistency reliability and validity have been reported to be acceptable for this version (Haratani et al. 1996). In this sample, Cronbach’s alpha coefficient was 0.86.

#### Outcome: psychological distress

Psychological distress was measured using the Japanese version of the K6 scale (Furukawa et al. 2008; Kessler et al. 2002). The K6 scale comprises six items measuring the levels of psychological distress, that is, feeling (1) nervous, (2) hopeless, (3) restless or fidgety, (4) so depressed that nothing could cheer you up, (5) everything was an effort, and (6) worthless, on a five-point scale ranging from 0 = None of the time to 4 = All of the time. The total score, ranging from 0 to 24, was calculated by summing the score for each item, with a higher score indicating greater psychological distress. The K6 scale was translated into Japanese language, and the internal consistency reliability and validity have been reported as acceptable for this version (Furukawa et al. 2008). In this sample, Cronbach’s alpha coefficient was 0.88.

#### Potential confounders

Demographic and occupational characteristics were considered potential confounders. A previous study showed that the association of psychosocial working conditions with mental health differs as a function of age (de Lange et al. 2006). Additionally, education has been reported to be associated with psychosocial working conditions (Lunau et al. 2015) as well as with mental health (Lorant et al. 2003). Furthermore, Adams et al. (1996) have suggested that the relationship between work and family can have an important effect on the employees’ well-being. Therefore, in addition to gender introduced earlier, age, education, and family size were included as confounding demographic characteristics. For occupational characteristics, previous studies have reported a gradient of psychosocial working conditions and health status across occupational classes (Kawakami et al. 2004; Marmot et al. 1991). Furthermore, shift workers have been reported to have poorer mental health (Vogel et al. 2012) as well as to be more exposed to unfavorable working conditions, including higher levels of JI, compared to day workers (Bøggild et al. 2001). Therefore, in addition to employment status introduced earlier, occupational class and work shift were included as confounding occupational characteristics.

Demographic characteristics were measured using the self-administered questionnaire. Age was used as a continuous variable. For education, the original classification in the self-administered questionnaire was five groups based on the previous study conducted in Japan (Kimura et al. 2016): graduate school, college, junior college, high school, and junior high school. However, the proportion of junior high school graduates was quite small; therefore, high school graduates and junior high school graduates were combined into one group. Family size was originally measured as a continuous variable. However, it may not necessarily have a linear association with psychological distress because each family size has different meanings (e.g., marital status, the necessity of child and/or family care, etc). Therefore, family size was treated as a categorical variable. In doing so, those who had a family of five or more were combined into one group.

For occupational characteristics, occupational class and work shift were measured using the self-administered questionnaire, whereas information on employment status was obtained from the personnel records of the surveyed company. Employment status was dichotomized into permanent and non-permanent employee. Occupational class was classified into nine groups using the original classification based on the 2008 version of the International Standardized Classification of Occupations (ISCO-08) major groups (International Labour Office 2012): manager, professional, technician, clerk, service and sales worker, craft and related trade worker, machine operator and assembler, laborer, and other. Work shift was classified into four groups using the original classification based on the previous study (Ohlander et al. 2015): day shift, shift work with night duty, shift work without night duty, and night shift.

#### Statistical analysis

Taking psychological distress (i.e., a total score for the K6 scale) as a dependent variable, hierarchical multiple regression analyses were conducted by gender and employment status in the following manner: potential confounders were initially entered into the model (Step 1) followed by the main effects of JI and RA (Step 2) and interaction term of JI × RA (Step 3). When the interaction effect of JI × RA in Step 3 emerged as significant, post hoc simple slope analyses were conducted at one standard deviation (SD) above/below the mean score of RA. In a series of analyses, *R*-squared (*R*^2^), adjusted *R*^2^, and *ΔR*^2^ (i.e., increase in *R*^2^ compared from the previous one) were calculated in each step to assess the model fit. In addition, residual analyses were conducted to estimate the amount of autocorrelation in the residuals using the Durbin-Watson statistic (ranging from 0 to 4.0 and a value of 2.0 means that there is no autocorrelation) and to check whether the standardized residuals are normally distributed. Prior to the analyses, total scores of JI and RA were mean-centered. It should be noted that the number of non-permanent male employees was quite small (*n* = 29), which might lead to reduced chance of detecting a true association due to low statistical power. Therefore, for non-permanent male employees, hierarchical multiple regression analyses were not conducted; instead, only information on demographic and occupational characteristics and scale scores was provided (see Table 1 described later). The level of significance was 0.05 (two-tailed). Statistical analyses were performed using IBM SPSS Statistics version 19 for Windows.

**Table 1:** Demographic and occupational characteristics, job insecurity, role ambiguity, and psychological distress of employees who participated in the present study (*n* = 2989)

Demographic and occupational characteristics	Men					Women				
	Permanent employee (*n* = 2155)		Non-permanent employee (*n* = 29)		*p* ^a^	Permanent employee (*n* = 333)		Non-permanent employee (*n* = 472)		*p* ^a^
	Mean (SD)	*n* (%)	Mean (SD)	*n* (%)		Mean (SD)	*n* (%)	Mean (SD)	*n* (%)	
Age	38.1 (11.1)		44.2 (9.67)		0.033	33.0 (10.1)		45.6 (6.98)		< 0.001
Education					0.266					0.002
Graduate school		302 (14.0)		1 (3.4)			19 (5.7)		9 (1.9)	
College		365 (16.9)		4 (13.8)			9 (2.7)		13 (2.8)	
Junior college		371 (17.2)		4 (13.8)			88 (26.4)		93 (19.7)	
High school or junior high school		1117 (51.8)		20 (69.0)			217 (65.2)		357 (75.6)	
Family size					0.209					< 0.001
One		587 (27.2)		3 (10.3)			80 (24.0)		17 (3.6)	
Two		316 (14.7)		5 (17.2)			59 (17.7)		73 (15.5)	
Three		366 (17.0)		7 (24.1)			47 (14.1)		71 (15.0)	
Four		543 (25.2)		10 (34.5)			71 (21.3)		170 (36.0)	
Five or more		343 (15.9)		4 (13.8)			76 (22.8)		141 (29.9)	
Occupational class					< 0.001					< 0.001
Manager		280 (13.0)		− (0.0)			− (0.0)		− (0.0)	
Professional		195 (9.0)		− (0.0)			18 (5.4)		1 (0.2)	
Technician		331 (15.4)		1 (3.4)			22 (6.6)		2 (0.4)	
Clerk		113 (5.2)		− (0.0)			117 (35.1)		41 (8.7)	
Service and sales worker		20 (0.9)		− (0.0)			− (0.0)		− (0.0)	
Craft and related trade worker		176 (8.2)		1 (3.4)			8 (2.4)		16 (3.4)	
Machine operator and assembler		620 (28.8)		7 (24.1)			22 (6.6)		48 (10.2)	
Laborer		237 (11.0)		15 (51.7)			70 (21.0)		157 (33.3)	
Other		183 (8.5)		5 (17.2)			76 (22.8)		207 (43.9)	
Work shift					< 0.001					< 0.001
Day shift		1332 (61.8)		− (0.0)			292 (87.7)		398 (84.3)	
Shift work with night duty		682 (31.6)		− (0.0)			18 (5.4)		− (0.0)	
Shift work without night duty		139 (6.5)		− (0.0)			16 (4.8)		− (0.0)	
Night shift		2 (0.1)		29 (100.0)			7 (2.1)		74 (15.7)	

Scale scores (range)	Mean (SD)	Estimate (SE)^b^	Mean (SD)	Estimate (SE)^b^	*p* ^b^	Mean (SD)	Estimate (SE)^b^	Mean (SD)	Estimate (SE)^b^	*p* ^b^
Job insecurity (JCQ) (4–17)	6.35 (1.86)	6.42 (0.09)	7.97 (2.15)	7.81 (0.38)	0.003	6.01 (1.64)	6.17 (0.14)	6.27 (1.57)	6.26 (0.13)	1.000
Role ambiguity (NIOSH-GJSQ) (6–42)	18.4 (5.49)	18.2 (0.27)	16.2 (7.27)	16.4 (1.14)	0.820	19.4 (5.46)	19.0 (0.42)	19.1 (5.58)	18.8 (0.38)	1.000
Psychological distress (K6) (0–24)	5.80 (4.64)	5.59 (0.23)	5.21 (4.92)	5.63 (0.98)	1.000	6.58 (4.96)	5.91 (0.36)	4.92 (4.43)	5.15 (0.33)	0.226

*JCQ* Job Content Questionnaire, *NIOSH-GJSQ* National Institute for Occupational Safety and Health Generic Job Stress Questionnaire

^a^Student’s *t*-test was used for continuous variables; Fisher’s exact test was used for categorical variables

^b^Bonferroni multiple comparison test was used while adjusting for demographic and occupational characteristics

## Results

During the survey period, 3461 employees completed the self-administered questionnaire (response rate = 95.3%). After excluding 472 employees who had at least one missing response on the questionnaires, the final sample comprised 2989 respondents (2184 men and 805 women: valid response rate = 82.3%). In the present sample, among non-permanent employees, men perceived significantly higher levels of JI compared to women, after adjusting for demographic and occupational characteristics (*p* for Bonferroni multiple comparison test < 0.001). Furthermore, among men, non-permanent employees perceived significantly higher levels of JI compared to permanent employees (*p* for Bonferroni multiple comparison test = 0.003). On the other hand, difference in RA or psychological distress between genders or employment status was not significant. Detailed demographic and occupational characteristics and scale scores by gender and employment status are shown in Table 1.

**Fig. 1:**
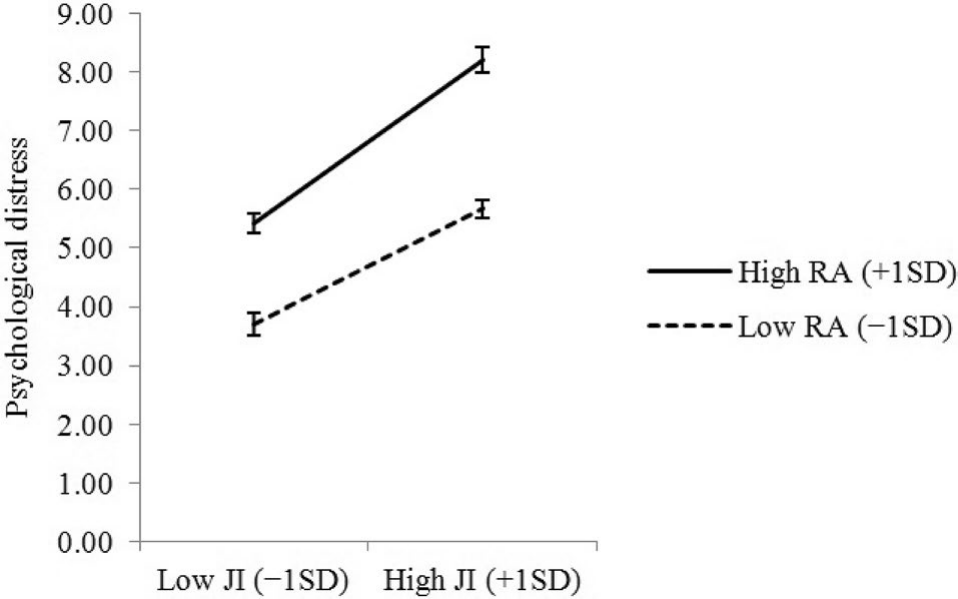
Interaction between job insecurity (JI) and role ambiguity (RA) on psychological distress among permanent male employees: post hoc simple slope analysis (2155 men)

Table 2 shows the results for permanent male employees. After adjusting for demographic and occupational characteristics (Step 2), both JI and RA had significant positive main effects on psychological distress (*β* = 0.268, *p* < 0.001 and *β* = 0.233, *p* < 0.001, respectively). When we added the interaction term of JI × RA in the model (Step 3), the main effects of JI and RA remained significant (*β* = 0.256, *p* < 0.001 and *β* = 0.229, *p* < 0.001, respectively). The interaction effect of JI × RA was also significant (*β* = 0.053, *p* = 0.010). Furthermore, the interaction term of JI × RA significantly contributed to the explanation of psychological distress (*ΔR*^2^ = 0.030, *p* = 0.010). Post hoc simple slope analyses showed that the simple slope of JI was greater at higher levels of RA (i.e., one SD above the mean) (*β* = 0.300, *p* < 0.001) rather than lower levels of RA (i.e., one SD below the mean) (*β* = 0.212, *p* < 0.001) (Fig. 1).

**Table 2:** Associations of demographic and occupational characteristics, job insecurity, and role ambiguity with psychological distress among permanent male employees: hierarchical multiple regression analysis (2155 men)

Standardized coefficient (*β*)	Step 1		Step 2		Step 3	
	Estimate	*p*	Estimate	*p*	Estimate	*p*
Age	− 0.096	< 0.001	− 0.112	< 0.001	− 0.113	< 0.001
Education (vs. high school or junior high school)						
Graduate school	− 0.014	0.603	− 0.012	0.622	− 0.011	0.643
College	− 0.035	0.178	− 0.023	0.333	− 0.023	0.332
Junior college	− 0.040	0.088	− 0.035	0.099	− 0.034	0.106
Family size (vs. one)						
Two	− 0.058	0.022	− 0.048	0.042	− 0.049	0.038
Three	− 0.033	0.211	− 0.018	0.444	− 0.017	0.468
Four	− 0.052	0.064	− 0.037	0.159	− 0.037	0.152
Five or more	− 0.052	0.054	− 0.038	0.126	− 0.039	0.119
Occupational class (vs. other)						
Manager	− 0.048	0.148	0.029	0.338	0.028	0.355
Professional	− 0.030	0.353	− 0.009	0.747	− 0.008	0.783
Technician	0.007	0.843	0.008	0.798	0.011	0.739
Clerk	0.009	0.742	0.035	0.157	0.036	0.149
Service and sales worker	− 0.017	0.466	− 0.007	0.736	− 0.007	0.724
Craft and related trade worker	− 0.005	0.866	0.002	0.951	0.005	0.861
Machine operator and assembler	0.033	0.410	0.019	0.606	0.022	0.544
Laborer	0.011	0.718	0.017	0.546	0.019	0.516
Work shift (vs. day shift)						
Shift work with night duty	− 0.083	0.004	− 0.095	< 0.001	− 0.096	< 0.001
Shift work without night duty	0.031	0.172	0.000	0.995	− 0.001	0.964
Night shift	− 0.009	0.663	− 0.019	0.349	− 0.015	0.463
Job insecurity			0.268	< 0.001	0.256	< 0.001
Role ambiguity			0.233	< 0.001	0.229	< 0.001
Job insecurity × role ambiguity					0.053	0.010

Model fit indices	Estimate	*p*	Estimate	*p*	Estimate	*p*
*R* ^2^	0.027	–	0.175	–	0.178	–
Adjusted *R*^2^	0.019	–	0.167	–	0.169	–
*ΔR* ^2^	0.027	< 0.001	0.148	< 0.001	0.030	0.010

Table 3 shows the results for permanent female employees. After adjusting for demographic and occupational characteristics (Step 2), both JI and RA had significant positive main effects on psychological distress (*β* = 0.173, *p* = 0.002 and *β* = 0.143, *p* = 0.009, respectively). When we added the interaction term of JI × RA in the model (Step 3), the main effects of JI and RA remained significant (*β* = 0.182, *p* = 0.001 and *β* = 0.145, *p* = 0.009, respectively). However, in contrast to permanent male employees, the interaction effect of JI × RA was not significant (*β*=-0.036, *p* = 0.500), and the interaction term of JI × RA did not significantly contribute to the explanation of psychological distress (*ΔR*^2^ = 0.001, *p* = 0.500).

**Table 3:** Associations of demographic and occupational characteristics, job insecurity, and role ambiguity with psychological distress among permanent female employees: hierarchical multiple regression analysis (333 women)

Standardized coefficient (*β*)	Step 1		Step 2		Step 3	
	Estimate	*p*	Estimate	*p*	Estimate	*p*
Age	− 0.278	< 0.001	− 0.273	< 0.001	− 0.269	< 0.001
Education (vs. high school or junior high school)						
Graduate school	0.101	0.084	0.101	0.074	0.104	0.068
College	0.039	0.477	0.013	0.806	0.014	0.792
Junior college	0.057	0.335	0.050	0.383	0.054	0.348
Family size (vs. one)						
Two	− 0.076	0.278	− 0.071	0.292	− 0.070	0.305
Three	− 0.082	0.230	− 0.089	0.181	− 0.090	0.175
Four	− 0.151	0.035	− 0.138	0.046	− 0.139	0.045
Five or more	− 0.035	0.633	− 0.029	0.682	− 0.030	0.665
Occupational class (vs. other)^a^						
Professional	− 0.066	0.310	− 0.062	0.317	− 0.060	0.337
Technician	− 0.064	0.354	− 0.067	0.314	− 0.069	0.304
Clerk	− 0.050	0.506	− 0.043	0.558	− 0.039	0.599
Craft and related trade worker	0.020	0.720	0.027	0.616	0.025	0.641
Machine operator and assembler	− 0.080	0.202	− 0.063	0.300	− 0.060	0.325
Laborer	− 0.079	0.235	− 0.087	0.178	− 0.085	0.191
Work shift (vs. day shift)						
Shift work with night duty	0.094	0.117	0.083	0.155	0.083	0.154
Shift work without night duty	0.047	0.391	0.033	0.531	0.031	0.553
Night shift	− 0.012	0.818	− 0.023	0.660	− 0.027	0.611
Job insecurity			0.173	0.002	0.182	0.001
Role ambiguity			0.143	0.009	0.145	0.009
Job insecurity × role ambiguity					− 0.036	0.500

Model fit indices	Estimate	*p*	Estimate	*p*	Estimate	*p*
*R* ^2^	0.142	–	0.204	–	0.205	–
Adjusted *R*^2^	0.096	–	0.156	–	0.154	–
*ΔR* ^2^	0.142	< 0.001	0.062	< 0.001	0.001	0.500

^a^There were no managers or service and sales workers among permanent female employees

Table 4 shows the results for non-permanent female employees. After adjusting for demographic and occupational characteristics (Step 2), both JI and RA had significant positive main effects on psychological distress (*β* = 0.336, *p* < 0.001 and *β* = 0.130, *p* = 0.004, respectively). When we added the interaction term of JI × RA in the model (Step 3), the main effects of JI and RA remained significant (*β* = 0.335, *p* < 0.001 and *β* = 0.129, *p* = 0.003, respectively). However, as with the case of permanent female employees, the interaction effect of JI × RA was not significant (*β* = 0.003, *p* = 0.952), and the interaction term of JI × RA did not significantly contribute to the explanation of psychological distress (*ΔR*^2^ = 0.000, *p* = 0.952).

**Table 4:** Associations of demographic and occupational characteristics, job insecurity, and role ambiguity with psychological distress among non-permanent female employees: hierarchical multiple regression analysis (472 women)

Standardized coefficient (*β*)	Step 1		Step 2		Step 3	
	Estimate	*p*	Estimate	*p*	Estimate	*p*
Age	− 0.037	0.454	− 0.035	0.435	− 0.036	0.434
Education (vs. high school or junior high school)						
Graduate school	0.016	0.730	0.052	0.231	0.052	0.232
College	0.000	0.998	− 0.024	0.596	− 0.024	0.597
Junior college	− 0.028	0.564	− 0.026	0.558	− 0.026	0.561
Family size (vs. one)						
Two	− 0.057	0.561	− 0.076	0.406	− 0.076	0.406
Three	− 0.068	0.489	− 0.073	0.420	− 0.073	0.420
Four	− 0.107	0.386	− 0.127	0.265	− 0.128	0.265
Five or more	− 0.138	0.249	− 0.138	0.210	− 0.139	0.210
Occupational class (vs. other)^a^						
Professional	− 0.049	0.296	− 0.028	0.514	− 0.028	0.514
Technician	0.089	0.058	0.080	0.064	0.080	0.064
Clerk	0.068	0.175	0.077	0.101	0.077	0.102
Craft and related trade worker	0.025	0.605	0.038	0.394	0.038	0.393
Machine operator and assembler	0.088	0.080	0.092	0.047	0.092	0.048
Laborer	0.100	0.048	0.106	0.024	0.106	0.025
Work shift (vs. day shift)^b^						
Night shift	− 0.042	0.394	− 0.040	0.380	− 0.040	0.381
Job insecurity			0.336	< 0.001	0.335	< 0.001
Role ambiguity			0.130	0.004	0.129	0.004
Job insecurity × role ambiguity					0.003	0.952

Model fit indices	Estimate	*p*	Estimate	*p*	Estimate	*p*
*R* ^2^	0.028	–	0.175	–	0.175	–
Adjusted *R*^2^	− 0.004	–	0.144	–	0.142	–
*ΔR* ^2^	0.028	0.600	0.147	< 0.001	0.000	0.952

^a^There were no managers or service and sales workers among non-permanent female employees

^b^All non-permanent female employees were day shift workers or night shift workers

For residual analyses of each group, the Durbin-Watson statistic ranged from 1.979 to 2.048 (i.e., very near to the optimum of 2.0) and the residual was normally distributed.

## Discussion

The present study demonstrated the significant main effects of JI and RA on psychological distress regardless of gender or employment status. The significant interaction effect of JI × RA was observed among permanent male employees in that the association of JI with psychological distress was greater when they perceived higher levels of RA. On the other hand, the interaction effect of JI × RA was not significant among permanent or non-permanent female employees. Non-permanent male employees were not included in the statistical analyses due to a small sample size.

In the present study, the significant main effect of JI on psychological distress was observed after adjusting for potential confounders (i.e., demographic and occupational characteristics) (Steps 2 and 3) regardless of gender or employment status. This finding is consistent with previous meta-analytic studies and a systematic review showing the association of JI with poor mental health, such as common mental disorders and depressive symptoms (Cheng and Chan 2008; Kim and von dem Knesebeck 2016; Stansfeld and Candy 2006; Sverke et al. 2002; Theorell et al. 2015). Similarly, the significant main effect of RA on psychological distress was observed after adjusting for potential confounders (Steps 2 and 3), regardless of gender or employment status. This finding is also consistent with a previous meta-analytic study and a systematic review showing the association of RA with depression and common mental health problems (Harvey et al. 2017; Schmidt et al. 2014). The present study replicated the findings from previous meta-analytic studies and systematic reviews on JI and RA in terms of psychological distress.

Furthermore, the interaction effect of JI × RA on psychological distress was significant after adjusting for potential confounders (Step 3) among permanent male employees. Post hoc simple slope analyses showed that the association of JI with psychological distress was greater when they perceived higher levels of RA. These findings support our hypothesis. Based on the COR theory introduced earlier (Hobfoll 1989), high RA (or low RC) may inhibit comprehension of employees’ work responsibilities, which may lead to a lack of clear expectations and increased uncertainty at the workplace. In such situation, the association of JI with psychological distress may be strengthened, because employees may be less likely to feel the control over an insecure situation (Tomas and Seršić 2015). On the other hand, this finding can also indicate that the association of RA with psychological distress was greater when they perceived higher levels of JI. According to Hackman and Oldham’s (1980) ‘job characteristics model’, the positive effect of psychosocial working conditions on work outcomes (e.g., job satisfaction and work motivation) occurs only when the employees’ concerns with job security are satisfied. Therefore, conversely, in the situation where such concerns are dissatisfied, the association of adverse working conditions, like high RA, with psychological distress may be strengthened. Such a modifying effect of JI on the association of RA with mental health outcomes should be examined more closely in the future.

In contrast to permanent male employees, the interaction effect of JI × RA on psychological distress was not significant among permanent or non-permanent female employees. This finding suggests that the extent of RA does not affect the association of JI with psychological distress, which does not support our hypothesis. The framework of gender-role ideology might explain the gender difference in the interaction effect of JI × RA, suggesting that work roles and breadwinning are more central to the identity of men, whereas family roles are more central to the identity of women (Barnett et al. 1995; Simon 1992). Since in the Japanese society, such a traditional gender-role ideology persists (Katsurada and Sugihara 2002), women may be less likely to be aware of the extent of ambiguity (or clarity) of their work roles in the context of JI compared to men. However, as the present sample of permanent and non-permanent female employees was small and drawn from one company, the finding remains uncertain and further research is needed to explore gender difference in the interaction effect of JI × RA using a broader sample.

In the present study, among non-permanent employees, men perceived significantly higher levels of JI compared to women. Similar tendency, although non-significant, was observed among permanent employees. The above-mentioned framework of gender-role ideology might also explain this finding. As introduced earlier, Charles and James (2005) argued that the gender-role ideology, suggesting that men are expected to be the primary breadwinners, is specifically linked to gender differences in experiences and perceptions of JI, with men feeling more insecure compared to women. Therefore, the present finding may reflect such a gender-role ideology that persists in the Japanese society (Katsurada and Sugihara 2002).

Furthermore, among women, the main effect of JI on psychological distress for non-permanent employees was about 1.8 times greater compared to permanent employees (*β* = 0.335 vs. 0.182 in Step 3). This finding is consistent with earlier findings of Koslowsky (1998), implying that the negative effects of two stressors (in the present case, JI and non-permanent employment) on mental health outcomes may strengthen each other in a multiplicative way. However, recent research has suggested that the association of JI with mental health outcomes is greater among permanent employees rather than non-permanent employees (e.g., De Witte and Näswall 2003). Bernhard-Oettel et al. (2005) have suggested that one of the reasons for such a mixed pattern of findings is that research on the interaction effect of JI with employment status typically does not consider the heterogeneous nature of non-permanent employees, such as contract duration, employment prospects, and preference for non-permanent employment. Therefore, to examine the interaction effect of JI with employment status on psychological distress more precisely, future studies should consider such heterogeneity of non-permanent employment.

The present study had some limitations. First, although the response rate in the present study was high, those who perceived higher levels of JI, RA, and psychological distress may have been less likely to participate in the present study. Second, although we adjusted for family size as a potential confounder, information on marital status was not obtained, since it is sensitive personal information. László et al. (2010) have suggested that JI has more deleterious effects on single persons than on married or cohabiting ones, as the social and the financial support from a spouse is likely to have an important protective effect. The present study could not completely eliminate such confounding bias. Third, as described earlier, the reliability of the JCQ JI scale was low in the present sample, which may have produced either overestimates or underestimates of substantive associations, although a previous cross-cultural study reported a similar level of reliability (Karasek et al. 1998). Fourth, the present sample was recruited from one manufacturing company with stable business conditions in Japan; therefore, the generalization of the present findings should be done with caution. Furthermore, as described earlier, non-permanent male employees could not be included in the statistical analyses due to a small sample size; therefore, a future study could try to replicate our findings with non-permanent male employees. Fifth, although a recent study on JI utilized a multilevel approach while considering its contextual effect (Låstad et al. 2016), the present study could not examine such an effect due to lack of information concerning the department of each participant. Sixth, causal inferences could not be made due to the cross-sectional nature of the study. The present findings seem to indicate that those who experienced higher levels of psychological distress may have been more likely to assess JI and/or RA as high. Finally, our main outcome was self-reported psychological distress; therefore, further studies could focus on more severe mental health outcomes, such as doctor-diagnosed depression.

Despite several limitations described above, the present study suggests that JI and RA have an interaction effect on psychological distress, at least among Japanese permanent male employees. JI is the most stressful aspect of the process leading to unemployment, having a worse effect on employees than unemployment does itself (Nella et al. 2015); therefore, to maintain and promote good mental health among employees, stronger employment measures and unemployment protection system should be developed at a national policy level (Uutela 2010). On the other hand, in the workplace, providing a clear description of job role may be effective in the reducing psychological distress associated with JI, especially among permanent male employees.

## Appendix: Job Content Questionnaire (JCQ) general job insecurity (JI) scale

**Table:**

Q1. My job security is good^†^
1 = Strongly disagree
2 = Disagree
3 = Agree
4 = Strongly agree
Q2. How steady is your work?^‡^
1 = Regular and steady
2 = Seasonal
3 = Frequent layoff
4 = Both seasonal and frequent layoff
Q3. During the past year, how often were you in a situation where you faced job loss of layoff?
1 = Never
2 = Once
3 = More than once
4 = Constantly
5 = Actually laid off
Q4. Sometimes people permanently lose jobs they want to keep. How likely is it that during the next couple of years you will lose present job with your employer?
1 = Not at all likely
2 = Not too likely
3 = Somewhat likely
4 = Very likely

^†^Reverse-scored item

^‡^Response options from 2 (seasonal) to 4 (both seasonal and frequent layoff) are converted to 4
